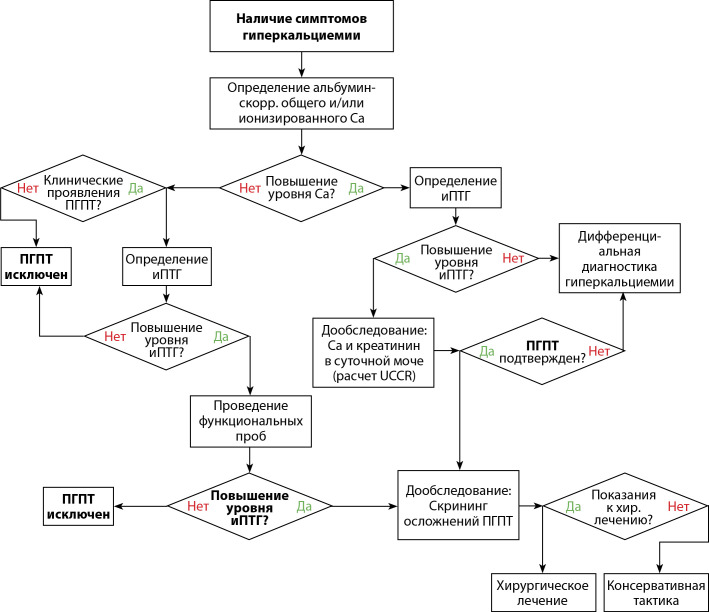# Клинические рекомендации по первичному гиперпаратиреозу, краткая версия

**DOI:** 10.14341/probl12801

**Published:** 2021-08-19

**Authors:** Н. Г. Мокрышева, А. К. Еремкина, С. С. Мирная, Ю. А. Крупинова, И. А. Воронкова, И. В. Ким, Д. Г. Бельцевич, Н. С. Кузнецов, Е. А. Пигарова, Л. Я. Рожинская, М. В. Дегтярев, Л. В. Егшатян, П. О. Румянцев, Е. Н. Андреева, М. Б. Анциферов, Н. В. Маркина, И. В. Крюкова, Т. Л. Каронова, С. В. Лукьянов, И. В. Слепцов, Н. Б. Чагай, Г. А. Мельниченко, И. И. Дедов

**Affiliations:** Национальный медицинский исследовательский центр эндокринологии; Национальный медицинский исследовательский центр эндокринологии; ООО «Сеть семейных медицинских центров»; Национальный медицинский исследовательский центр эндокринологии; Национальный медицинский исследовательский центр эндокринологии; Национальный медицинский исследовательский центр эндокринологии; Национальный медицинский исследовательский центр эндокринологии; Национальный медицинский исследовательский центр эндокринологии; Национальный медицинский исследовательский центр эндокринологии; Национальный медицинский исследовательский центр эндокринологии; Национальный медицинский исследовательский центр эндокринологии; Национальный медицинский исследовательский центр эндокринологии; Международный медицинский центр «СОГАЗ»; Национальный медицинский исследовательский центр эндокринологии; Эндокринологический диспансер Департамента здравоохранения города Москвы; Эндокринологический диспансер Департамента здравоохранения города Москвы; Московский областной научно-исследовательский клинический институт им. М.Ф. Владимирского; Национальный медицинский исследовательский центр им. В.А. Алмазова; Ростовский государственный медицинский университет; Санкт-Петербургский многопрофильный центр; Ставропольский государственный медицинский университет; Национальный медицинский исследовательский центр эндокринологии; Национальный медицинский исследовательский центр эндокринологии

**Keywords:** околощитовидные железы, первичный гиперпаратиреоз, паратгормон, витамин D, остеопороз, патологические переломы, нефролитиаз

## Abstract

Первичный гиперпаратиреоз (ПГПТ) — эндокринное заболевание, характеризующееся избыточной секрецией паратиреоидного гормона (ПТГ) при верхненормальном или повышенном уровне кальция крови вследствие первичной патологии околощитовидных желез (ОЩЖ). ПГПТ проявляется полиорганными нарушениями различной степени выраженности и, как следствие, может приводить к существенному снижению качества жизни, инвалидизации пациентов, повышенному риску преждевременной смерти. Гиперкальциемия и катаболическое действие ПТГ на клетки различных органов рассматриваются как основные патогенетические механизмы развития осложнений при ПГПТ. За последние десятилетия отмечено резкое увеличение заболеваемости ПГПТ, в основном за счет выявления мягких форм заболевания, что прежде всего обусловлено совершенствованием методов диагностики и внедрением скрининга кальциемии в странах Северной Америки, Западной Европы и Азии. Увеличение выявляемости ПГПТ, многообразие клинических проявлений и форм этого заболевания привело к тому, что эти пациенты стали попадать в поле зрения различных специалистов — терапевтов, ревматологов, урологов, нефрологов, кардиологов и других врачей. В статье изложены основные тезисы клинических рекомендаций по ведению пациентов с ПГПТ, утвержденных Минздравом России в 2020 г. Изложены алгоритмы лабораторной и инструментальной диагностики, дифференциально-диагностических проб, рассмотрены вопросы хирургической и консервативной тактики ведения, динамического наблюдения за пациентами с данной нозологией. Отдельно выделены рекомендации для особых групп пациентов — наследственные формы ПГПТ, злокачественное поражение ОЩЖ, течение ПГПТ при беременности.

## Abstract (en)

Primary hyperparathyroidism (PHPT) is an endocrine disorder of parathyroid glands characterized by excessive secretion of parathyroid hormone (PTH) with an upper normal or elevated blood calcium level. Classical PHPT refers to a symptomatic, multi-system disorder, wich can lead to a significant decrease in the quality of life, disability of patients, and even an increased risk of premature death. Hypercalcemia and the catabolic effect of PTH on various cells are considered as the main pathogenetic mechanisms of the PHPT associated complications. In the last two decades, there has been an increase in the incidence of PHPT, mainly due to the mild forms of the disease, primarily due to the routine calcium screening in North America, Western Europe and, Asia. High prevalence of the disease, as well as the variety of clinical manifestations, cause the attention of different specialists - physicians, rheumatologists, urologists, nephrologists, cardiologists and other doctors. This review cover the main issues of Russian guidelines for the management of PHPT, approved in 2020, including laboratory and instrumental methods, differential diagnosis, surgical and conservative approach, short-term and long-term follow-up. This guidelines also include the recommendations for special groups of patients with hereditary forms of PHPT, parathyroid carcinoma, PHPT during pregnancy.

## ВВЕДЕНИЕ

Первичный гиперпаратиреоз (ПГПТ) — эндокринное заболевание, характеризующееся избыточной секрецией ПТГ при верхне-нормальном или повышенном уровне кальция крови вследствие первичной патологии околощитовидных желез (ОЩЖ). ПГПТ проявляется полиорганными нарушениями различной степени выраженности и, как следствие, может приводить к существенному снижению качества жизни, инвалидизации пациентов, повышенному риску преждевременной смерти [1]. ПГПТ в 85–90% случаев обусловлен солитарной аденомой ОЩЖ, в 5–10% случаев — множественными аденомами или гиперплазией нескольких/всех ОЩЖ; в 1% — раком ОЩЖ. В 90–95% случаев ПГПТ является спорадическим, около 5–10% составляют наследственные формы, которые проявляются изолированной патологией ОЩЖ или протекают в сочетании с другими компонентами генетически детерминированных синдромов [2].

Патогенез опухолей ОЩЖ изучен недостаточно, в литературе обсуждается влияние некоторых протоонкогенов и генов супрессоров опухолевой активности на развитие новообразований. В качестве предположительных механизмов запуска гиперплазии с последующей трансформацией в аденому ОЩЖ рассматриваются хронический дефицит витамина D и усиление его инактивации в печени [3]. В ряде случаев спорадического ПГПТ определяются мутации в генах MEN1, CDC73, CASR, реже CDKIs, EZH2, POT1 [4].

ПГПТ, сопровождающийся множественными гиперплазиями или аденомами ОЩЖ, часто связан с наследственными синдромами. Синдром множественных эндокринных неоплазий 1 типа (МЭН-1) ассоциирован с мутациями в гене-супрессоре опухолевого роста MEN1; синдром МЭН-2 — с мутациями протоонкогена RET; синдром МЭН-4 развивается вследствие мутации ингибитора циклинзависимой киназы CDNK1B [5]. Синдром гиперпаратиреоза с опухолью нижней челюсти (HPT-JT) ассоциирован с мутациями в гене CDC73, кодирующем белок парафибромин [6]. Семейный изолированный гиперпаратиреоз (FIHP) характеризуется развитием опухолей одной или нескольких ОЩЖ и отсутствием других новообразований эндокринных и неэндокринных органов, иногда может представлять собой неполный вариант других синдромов (МЭН-1, HPT-JT). FIHP может быть обусловлен мутациями в генах MEN1, CASR и CDC73, но чаще генетическая причина остается неизвестной [7]. Семейная гипокальциурическая гиперкальциемия (FHH) — генетически гетерогенное заболевание, обусловленное мутациями в генах CASR, Gα11, AP2S1, требует проведения дифференциальной диагностики с ПГПТ [5].

За последние десятилетия произошли существенные изменения в представлениях об эпидемиологии заболевания. Отмечено резкое увеличение выявляемости ПГПТ, в том числе за счет бессимптомных форм, не сопровождающихся высокой гиперкальциемией. Указанные изменения обусловлены прежде всего появлением автоматических биохимических анализаторов и активным внедрением повсеместного определения уровня кальция в странах Северной Америки, Западной Европы и Китае. В общей популяции распространенность ПГПТ составляет в среднем 0,86–1% [8]. ПГПТ может встречаться во всех возрастных группах, включая детей и подростков. Однако совокупность фактических данных свидетельствует о том, что частота возникновения ПГПТ увеличивается с возрастом, и средний возраст на момент постановки диагноза составляет 54–59 лет [9]. Большинство пациентов при спорадическом ПГПТ — женщины в постменопаузе с развитием заболевания в течение первого десятилетия после наступления менопаузы. Соотношение мужчин и женщин в среднем 1:3 [9].

У большей части пациентов в Российской Федерации гиперкальциемия диагностируется отсроченно, поскольку определение содержания кальция не входит в общетерапевтический биохимический анализ крови. Это создает предпосылки для позднего выявления гиперпаратиреоза. В Российской Федерации широкомасштабных эпидемиологических исследований не проводилось. По результатам анализа 1914 пациентов с ПГПТ (Российский регистр пациентов с первичным гиперпаратиреозом) на декабрь 2017 г. наблюдается возрастание распространенности заболевания по г. Москве. В 2017 г. она составила 13 случаев на 100 000 взрослого населения (2016 г. — 5,6 случая, на 2010 г. — 4 случая на 100 000 взрослого населения). По Московской области распространенность ПГПТ на декабрь 2017 г. составила 3,4 случая на 100 000 взрослого населения (по сравнению с 2014 г. — 0,25 случаев). Несмотря на повышение распространенности, полученные данные не соответствуют частоте гиперкальциемии, обнаруженной по данным пилотного скрининга уровня кальция среди взрослого населения. В основном преобладали манифестные формы ПГПТ — в 67% случаев, бессимптомное течение заболевания определялось в 33%, в то время как в странах Европы, Северной Америки уже к 2004 г. частота манифестных форм не превышала 20%.

## КЛАССИФИКАЦИЯ ПЕРВИЧНОГО ГИПЕРПАРАТИРЕОЗА

Симптомный (манифестный) ПГПТ характеризуется наличием «классических» проявлений заболевания, к которым относят костные (остеопороз, низкотравматичные переломы и фиброзно-кистозный остеит) и висцеральные нарушения (нефролитиаз, язвенная болезнь верхних отделов слизистой желудочно-кишечного тракта (ЖКТ)). В настоящее время к пациентам с бессимптомным ПГПТ (ранее классифицировали как мягкую форму) относят лиц, не имеющих специфических проявлений заболевания, при этом диагностика заболевания, как правило, происходит на этапе рутинного скрининга кальция. Ряд исследований свидетельствует о возможности длительного доброкачественного течения бессимптомного ПГПТ у большинства пациентов. Однако у некоторых пациентов с течением времени отмечается прогрессирование заболевания с развитием специфической симптоматики [9].

Наиболее часто диагностируется гиперкальциемический вариант ПГПТ, характеризующийся повышением уровня кальция сыворотки крови в сочетании с повышенным (редко высоко-нормальным) уровнем интактного ПТГ (иПТГ). Однако ПГПТ не всегда сопровождается повышением уровня кальция крови выше верхней границы референсного диапазона. Нормокальциемия может быть транзиторной при гиперкальциемическом варианте и стойкой при нормокальциемическом варианте заболевания. Нормокальциемический вариант ПГПТ (нПГПТ) характеризуется неизменно верхненормальным уровнем общего и ионизированного кальция (Са++) в сыворотке крови в сочетании со стойким повышением уровня иПТГ, в отсутствие очевидных причин вторичного гиперпаратиреоза (ВГПТ) (дефицит витамина D, патология печени и почек, синдром мальабсорбции, гиперкальциурии и др.) [9][10]. Классификация ПГПТ представлена на рис. 1.

**Figure:**
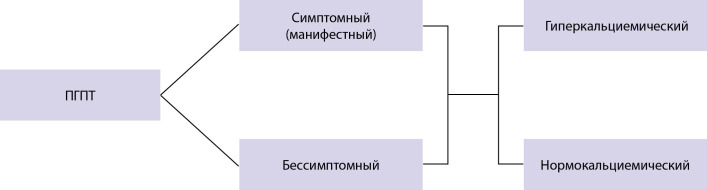
Рисунок 1. Классификация ПГПТ

## КРИТЕРИИ ПОСТАНОВКИ ДИАГНОЗА ПЕРВИЧНОГО ГИПЕРПАРАТИРЕОЗА

-стойкое сохранение показателей альбумин-скорректированного и Са++ в референсном диапазоне за весь период наблюдения при повышенном уровне иПТГ (минимум двукратное определение в интервале 3–6 мес);

-исключение возможных вторичных причин гиперпаратиреоза (прежде всего дефицита/недостаточности витамина D (25(ОН)D ≤30 нг/мл) и ХБП (СКФ) ≤60 мл/мин));

-отсутствие гиперкальциурии.

УУР С (УДД — 4)

ЖАЛОБЫ И АНАМНЕЗ

-клинические симптомы гиперкальциемии, включая инсипидарный синдром (полиурия/никтурия/полидипсия, не обусловленные сахарным или несахарным диабетом); тошноту, рвоту, снижение аппетита, дегидратацию [28][29];

УУР С (УДД — 4)

-остеопороз или предшествующие низкотравматичные переломы в анамнезе (особенно переломы шейки бедра, переломы дистального отдела костей предплечья), клинические проявления фиброзно-кистозного остеита, включая деформации скелета, боли в ребрах [30–34];

УУР В (УДД — 3)

-нефролитиаз (особенно рецидивирующий), нефрокальциноз [34–40];

УУР В (УДД — 2)

-рецидивирующая язвенная болезнь желудка или двенадцатиперстной кишки, калькулезный панкреатит [41–43].

УУР C (УДД — 4)

Комментарии: клинические проявления ПГПТ в большинстве случаев обусловлены гиперкальциемией. Легкая гиперкальциемия (общий кальций <3 ммоль/л (12 мг/дл)) может длительно оставаться бессимптомной или проявляться в виде неспецифических жалоб, таких как общая и мышечная слабость, утомляемость, снижение эмоционального фона. Умеренная гиперкальциемия (общий кальций сыворотки крови >3–3,5 ммоль/л (12–14 мг/дл)) при хроническом течении может протекать с минимальной симптоматикой. Быстро прогрессирующая гиперкальциемия может сопровождаться ухудшением состояния в виде появления таких симптомов, как полиурия, полидипсия, дегидратация, снижение аппетита, тошнота, мышечная слабость. У пациентов с тяжелой гиперкальциемией (общий кальций сыворотки крови >3,5 ммоль/л (14 мг/дл)) отмечается высокий риск гиперкальциемического криза [44][45]. При сборе анамнеза необходимо учитывать прием препаратов, влияющих на фосфорно-кальциевый обмен таб. 1.

Нефролитиаз — одно из основных осложнений ПГПТ. В 10% случаев симптомный нефролитиаз при ПГПТ характеризуется рецидивирующим течением с частыми приступами почечной колики [40][46]. Распространенность ПГПТ среди больных с мочекаменной болезнью может значимо превышать общепопуляционные значения и достигать 3,2–5% [35–39]. Чаще стали диагностироваться «молчащие» формы заболевания (бессимптомный нефролитиаз/нефрокальциноз), выявляемые при проведении визуализирующих методов исследования (ультразвуковое исследование (УЗИ), компьютерная томография (КТ) почек) [47].

В настоящее время фиброзно-кистозный остеит как наиболее тяжелое костное проявление ПГПТ диагностируется значимо реже, тем не менее пациенты могут сообщать о множественных перенесенных низкотравматичных переломах, деформациях скелета и изменении походки. При бессимптомном течении ПГПТ патологические изменения скелета верифицируются, как правило, при снижении минеральной плотности кости (МПК) по результатам рентгенденситометрии [2][48].

Необходим тщательный сбор анамнеза касательно других симптомов, ассоциированных с ПГПТ, включая язвенную болезнь желудка и двенадцатиперстной кишки, гастроэзофагеальную рефлюксную болезнь, острый панкреатит, чаще ассоциированные с умеренной и тяжелой гиперкальциемией [49].

Проявления со стороны мышечной системы, включающие проксимальную мышечную слабость и атрофию, в настоящее время редки, но многие пациенты с ПГПТ предъявляют субъективные жалобы на повышенную утомляемость и генерализованную слабость [50–52]. Ранее к классическим психическим проявлениям ПГПТ относили тяжелое нарушение когнитивных функций и сознания, острый психоз, но они, как правило являются следствием тяжелой гиперкальциемии. В настоящее время все чаще сообщается об умеренных психических расстройствах, таких, как усталость, депрессия, эмоциональная лабильность, нарушения сна, ухудшение памяти и неспособность сконцентрироваться.

Необходим тщательный сбор персонального и семейного анамнеза при подозрении на синдромальные формы эндокринопатий.

## ФИЗИКАЛЬНОЕ ОБСЛЕДОВАНИЕ

УУР С (УДД — 5)

## ЛАБОРАТОРНЫЕ ДИАГНОСТИЧЕСКИЕ ИССЛЕДОВАНИЯ

Диагноз ПГПТ основывается только на данных лабораторного обследования.

УУР С (УДД — 4)

Комментарии: при подозрении на ПГПТ первоначально рекомендуется определение уровня альбумин-скорректированного кальция крови, при этом гиперкальциемия должна быть подтверждена более чем в одном измерении, прежде чем пациенту будет назначено расширенное обследование. Корректировка кальция на уровень альбумина крови необходима с целью исключения ложно завышенных или ложно заниженных показателей кальциемии при изменении концентрации плазменных белков [55]. Коррекция общего кальция рекомендуется при уровне альбумина менее 40 г/л и более 45 г/л. Ложных результатов можно также избежать путем прямого определения Са++ [12]. Этот показатель менее вариабельный, однако для его определения необходимо специальное оборудование — анализатор с использованием ион-селективных электродов, доступность которого в клинических лабораториях может быть лимитирована.

Формулы для расчета альбумин-скорректированного кальция:

общий кальций плазмы (ммоль/л) = измеренный уровень общего кальция плазмы (ммоль/л) + 0,02 × (40 - измеренный уровень альбумина плазмы (г/л))общий кальций плазмы (мг/дл) = измеренный уровень общего кальция плазмы (мг/дл) + 0,8 × (4 - измеренный уровень альбумина плазмы (г/дл))коэффициент пересчета: [кальций] мг/дл × 0,25 = [кальций] ммоль/л

УУР В (УДД — 3)

Комментарии: для пациентов с ПГПТ уровень иПТГ, как правило, повышен или находится на верхней границе референсного диапазона [12][13]. В случае приема лекарственных средств, способных влиять на уровень кальция и/или иПТГ, проведение лабораторных тестов рекомендуется после отмены препаратов (табл. 1). Уровни иПТГ, находящиеся в нижнем диапазоне нормальных значений (<25–30 пг/мл), более характерны для других состояний, ассоциированных с развитием гиперкальциемии. Прежде всего необходимо исключить паранеопластические процессы как вторую наиболее частую причину повышения уровня кальция крови.

**Table:** Таблица 1. Препараты, влияющие на показатели фосфорно-кальциевого обмена

Наименование препарата	Механизм действия
Гидрохлоротиазид**	Увеличение реабсорбции Ca++ в почках
Препараты лития	Повышение порога чувствительности ОЩЖ с увеличением уровня кальция и иПТГ крови
Бисфосфонаты (M05BA)	Развитие гипокальциемии вследствие угнетения процессов костной резорбции
деносумаб**	Развитие гипокальциемии вследствие угнетения процессов костной резорбции
цинакальцет**	Кальцимиметическое действие со снижением концентрации иПТГ и, как следствие, уменьшение содержания кальция в сыворотке крови
терипаратид**	Рекомбинантный человеческий ПТГ, подобно эндогенному гормону увеличивает кишечную абсорбцию и канальцевую реабсорбцию кальция, потенциально может определяться в рамках гормонального анализа
Витамин D и его производные	Риск передозировки при уровне 25(ОН)D более 100 нг/млНазначение активных альфакальцидола** или кальцитриола** в дозе 0,5 мкг в сутки и более может способствовать развитию гиперкальциемии

У 1/3 пациентов выявляется гипофосфатемия за счет реципрокного отношения между кальцием и фосфором. Также для ПГПТ характерны повышенные или верхненормальные уровни общей щелочной фосфатазы и более специфичных маркеров костного метаболизма [10].

УУР С (УДД — 4)

Креатинин сыворотки считается важным диагностическим инструментом в когорте пациентов с ПГПТ, так как почки являются одним из основных органов регуляции обмена кальция и фосфора в организме. Клиренсовые радиоизотопные методики — золотой стандарт в определении СКФ, однако стоимость и технические сложности резко лимитируют их широкое применение. В клинической практике для расчета СКФ могут быть использованы простые способы расчета клиренса креатинина, позволяющие обойтись без суточного сбора мочи [64].

УУР В (УДД — 3)

Комментарии: низкий уровень 25(ОН)D при ПГПТ ассоциирован с более высоким уровнем иПТГ и, как следствие уровнем кальция сыворотки крови, более низкой МПК в кортикальной зоне костей и более высокими показателями маркеров костного обмена, более частым развитием фиброзного остеита и большим весом аденомы ОЩЖ [65–67]. Оценка статуса витамина D проводится путем определения 25(ОН) витамина D в крови, что является наиболее доступным и надежным методом лабораторной диагностики [54].

УУР В (УДД — 2)

Комментарии: измерение кальция в суточной моче по отношению к экскреции креатинина необходимо с целью дифференциальной диагностики ПГПТ и семейной гипокальциурической гиперкальциемии (FHH). FHH — это редкое наследственное заболевание с аутосомно-доминантным типом наследования, вызванное дефектом кальций-чувствительных рецепторов (CaSR) в почках и ОЩЖ. Диагноз FHH может быть заподозрен при сочетании гиперкальциемии, нормального или несколько повышенного уровня иПТГ и относительной гипокальциурии. Для дифференциальной диагностики используется расчет отношения почечного клиренса кальция к клиренсу креатинина (UCCR), который обычно при FHH составляет менее 0,01.

Формула для расчета почечного клиренса кальция к клиренсу креатинина [11]

CaCl/CrCl = [Cau x Crs]/[Cru x Cas]CaCl — клиренс кальция, CrCl — клиренс креатинина, Cau — концентрация кальция в моче (ммоль/л), Crs — концентрация креатинина в сыворотке крови (мкмоль/л), Cru — концентрация креатинина в моче (мкмоль/л), Cas — концентрация кальция в сыворотке крови (ммоль/л)

Дополнительной ценностью данного анализа является определение суточной кальциурии, как показателя риска возникновения или прогрессирования нефролитиаза. При выявлении суточной экскреции кальция выше 10 ммоль/сутки показано проведение хирургического лечения ПГПТ. Необходимо отметить, что исследование кальция в моче не информативно при снижении функции почек (СКФ<60 мл/мин/1,73м2).

УУР С (УДД — 4)

Комментарии: сочетание повышенного показателя иПТГ в крови с нормальным уровнем сывороточного кальция остается актуальной клинической проблемой. В целях дифференциальной диагностики между нормокальциемическим вариантом ПГПТ и ВГПТ, возникшим в результате недостаточности витамина D или при других состояниях [73, 74]. необходимо проведение функциональных проб (табл. 2) [15][16]. У пациентов с ПГПТ прием препаратов витамина D и его производных и/или гидрохлоротиазида** провоцирует развитие гиперкальциемии при сохранении повышенного уровня иПТГ, а у пациентов с ВГПТ–снижение/нормализацию уровня иПТГ при нормальном уровне кальция в крови [73][75]. При наличии гиперкальциурии целесообразно проведение пробы с тиазидами.

**Table:** Таблица 2. Функциональные пробы для дифференциальной диагностики между первичной нормокальциемической и вторичной формами гиперпаратиреоза

Наименование препарата	Доза препарата	Интерпретация результатов	Сроки проведения
Колекальциферол** [73][76][77]	Пациентам с установленным дефицитом 25(ОН) витамина D c целью достижения целевого уровня витамина D более 30 нг/мл (75 нмоль/л)	Подтверждение ПГПТ: отсутствие снижения уровня иПТГ и в некоторых случаях появление гиперкальциемии.Исключение ПГПТ: нормализация уровня иПТГ при уровне кальция в крови в референсном диапазоне1	До достижения целевого уровня 25 (ОН) витамина D
Альфакальцидол** [70]	Пациентам при нарушении обмена кальция и фосфора вследствие нарушения эндогенного синтеза 1,25 дигидроксиколекальциферола (активного метаболита витамина D)1 мкг в сутки в течение 5–7 дней, анализ крови на 5–7-й день на кальций общий, альбумин, иПТГ.При отсутствии гиперкальциемии продолжение пробы до 1 месяца1	Подтверждение ПГПТ: отсутствие снижения уровня иПТГ и часто развитие гиперкальциемииИсключение ПГПТ: нормализация уровня иПТГ при уровне кальция в крови в референсном диапазоне*	От 5–7 дней до 1 месяца
Тиазиды:#Гидрохлоротиазид** [90][69]	По 25 мг 2 раза в сутки в течение 2 недель.Анализ крови на ПТГ на 15-й день	Подтверждение ПГПТ: отсутствие нормализации иПТГ и возможно, повышение уровня кальция сыворотки крови (нормализация через несколько суток после отмены препарата). Исключение ПГПТ: нормализация уровня иПТГ	2 недели

## ИНСТРУМЕНТАЛЬНЫЕ ДИАГНОСТИЧЕСКИЕ ИССЛЕДОВАНИЯ

Диагноз ПГПТ устанавливается исключительно на основании результатов лабораторного обследования, данные визуализирующих методов исследования не должны рассматриваться как способ дифференциальной диагностики различных форм гиперпаратиреоза. Применение визуализирующих методов исследования обязательно только для подготовки пациентов к хирургическому лечению верифицированного гиперпаратиреоза. Цель предоперационной топической диагностики образования ОЩЖ при ПГПТ — подготовка к селективной паратиреоидэктомии. Для планирования эффективного хирургического лечения требуется информация о точном месте расположения образования ОЩЖ.

УУР А (УДД — 1)

Комментарии: чувствительность УЗИ в случае солитарного образования ОЩЖ, по различным данным, варьируется от 76 до 91% и во многом зависит от квалификации специалиста [78–81]. Специфичность метода может достигать 96% [82–84]. Установлена высокая положительная прогностическая значимость и диагностическая точность УЗИ ОЩЖ (93,2% и 88% cоответственно) [78]. Аденома ОЩЖ, как правило, представляет собой образование округлой или овальной формы, гипоэхогенной структуры, очерченное изоэхогенной линией и контрастирующее с вышележащей гиперэхогенной тканью щитовидной железы. В ряде случаев могут визуализироваться кальцинаты и определяться кистозная дегенерация. В последнее время активно стали использовать УЗИ с контрастированием. Метод обеспечивает количественную и качественную оценку васкуляризации микрососудистого русла желез, что позволяет идентифицировать опухоли ОЩЖ. УЗИ с контрастированием обладает особыми преимуществами при наличии сопутствующего зоба, шейной лимфаденопатии, а также при полигландулярном поражении ОЩЖ даже после предшествующих хирургических вмешательств в области шеи [85].

Преимущества метода: отсутствие лучевой нагрузки; относительно низкая стоимость и высокая доступность исследования; возможность выявления сопутствующей патологии ЩЖ. Недостатки метода: значимая вариабельность диагностической точности метода в зависимости от квалификации специалиста; ложноположительные результаты за счет узловых образований ЩЖ или лимфатических узлов; значимые ограничения в проведении исследования при атипичной локализации ОЩЖ (например, за грудиной, в позадипищеводном пространстве).

УУР А (УДД — 1)

Комментарии: длительное время стандартной методикой оставалась двухмерная планарная сцинтиграфия. В настоящее время все чаще используется трехмерная однофотонная эмиссионная компьютерная томография (ОФЭКТ) и гибридная технология ОФЭКТ/КТ, сочетающая в себе функциональную чувствительность ОФЭКТ с высокой анатомической детализацией многослойной КТ. Чувствительность метода может варьировать в широком диапазоне, от 54 до 96% [88], в среднем составляя около 88% в случае солитарного образования ОЩЖ [80]. Множественное поражение ассоциировано со значимым снижением чувствительности: до 33% при наличии двух аденом ОЩЖ и до 44% в случае гиперплазии 4 ОЩЖ [80]. Отсутствие визуализации аденом ОЩЖ при проведении сцинтиграфии при ПГПТ ассоциировано с повышенным риском полигландулярного поражения. Объединенная чувствительность и положительная прогностическая значимость для сцинтиграфии с технеция [ 99mTс ] сестамиби с ОФЭКТ составляют 79% (49–91%) и 91% (84–96%) соответственно [78]. Для ОФЭКТ/КТ отмечена большая чувствительность метода (86%, ДИ 81–90) при сравнении с ОФЭКТ (74%, ДИ 66–82) и двухмерной планарной сцинтиграфией (70%, ДИ 61–80), кроме того, данный метод имеет преимущество перед другими методиками в топической диагностике эктопированных образований [87].

Преимущества метода: определение локализации как типично расположенных, так и эктопированных образований ОЩЖ, в том числе в средостении (основываясь на специфичности метаболизма паратиреоидной ткани). Недостатки метода: значимая вариабельность диагностической точности исследования в зависимости от центра (опыта специалистов, технической оснащенности — планарная гамма-камера, ОФЭКТ, ОФЭКТ-КТ), где проводится исследование; возможные ложноположительные и ложноотрицательные результаты при наличии сопутствующих заболеваний щитовидной железы (аутоиммунный тиреоидит, многоузловой зоб, диффузно-токсический зоб, рак щитовидной железы); снижение чувствительности метода при поражении нескольких ОЩЖ; лучевая нагрузка.

Комбинация ОФЭКТ-КТ с технеция [ 99mTс ] сестамиби и УЗИ ОЩЖ экспертного класса на дооперационном этапе диагностики ПГПТ повышают чувствительность до 95–98%. В случае множественного поражения ОЩЖ чувствительность данной комбинации остается невысокой и составляет 30–60% [89].

УУР А (УДД — 1)

УУР А (УДД — 2)

Комментарии: в спорных случаях, при расхождении результатов УЗИ и сцинтиграфии с технеция [ 99mTс ] сестамиби, применяется КТ шеи с внутривенным болюсным контрастированием. Традиционная КТ с контрастным усилением позволяет достаточно точно оценить размеры и локализацию образований ОЩЖ как в случае их типичного расположения, так и при наличии измененных эктопированных ОЩЖ, в том числе в средостении. Диагностическая чувствительность мультиспиральной КТ (МСКТ) сильно вариабельна и может составлять 46–87% [94]. Недостатками метода являются лучевая нагрузка, потенциальная нефротоксичность контрастного вещества и, соответственно ограниченное применение у пациентов с хронической болезнью почек (ХБП). Кроме того, исследование со стандартным шагом 5 мм возможно только при наличии желез массой 5 г и более, ошибка в данном случае не превышает 5%. В остальных случаях необходим более мелкий шаг снимков, что еще больше увеличивает лучевую нагрузку [95].

4D КТ демонстрирует многообещающие результаты в визуализации ОЩЖ, однако в настоящее время исследование малодоступно. Чувствительность метода 4D КТ составляет в среднем 89%, а положительная прогностическая значимость достигает 93,5% [78]. Относительно высокая чувствительность метода сохраняется при множественном поражения ОЩЖ — 62,5–85,7% [96–98]. Основными недостатками 4D-КТ являются высокая стоимость, значительное увеличение радиационного облучения, резко ограниченная доступность.

Проведение МРТ возможно для установления локализации патологически измененных ОЩЖ, однако данный метод обладает рядом недостатков: высокая стоимость, меньшая чувствительность (по разным данным, 43–71%, трудности в интерпретации полученных данных, что связано с возникновением артефактов при дыхательных движениях. Возможным преимуществом является выявление эктопированных в средостение ОЩЖ, но по точности данный метод уступает МСКТ [92][93].

Применение ПЭТ рекомендуется в отдельных случаях у пациентов с персистенцией заболевания или с рецидивом ПГПТ при отсутствии визуализации ОЩЖ с помощью других методов [91]. Однако данная методика является дорогостоящей и малодоступной, что препятствует ее широкому клиническому применению [99].

УУР А (УДД — 2)

Комментарии: данный метод полезен в случае необходимости дифференциальной диагностики образований ОЩЖ и узловых образований ЩЖ при отсутствии четкого подтверждения интратиреоидного расположения аденомы ОЩЖ по данным визуализирующих методик. Определение уровня иПТГ проводится с помощью стандартных наборов. Как правило, уровень иПТГ более 500 пг/мл соответствует патологически измененной ОЩЖ [103]. Цитологическое исследование ОЩЖ не проводится ввиду сложности дифференциальной диагностики с фолликулярными опухолями щитовидной желез. Кроме того, цитологическое исследование не позволяет дифференцировать доброкачественное от злокачественного поражения ОЩЖ [100–102].

ИНЫЕ ДИАГНОСТИЧЕСКИЕ ИССЛЕДОВАНИЯ

УУР С (УДД — 5)

Комментарии: рентгенологическая картина костных поражений вследствие ПГПТ включает в себя субпериостальную резорбцию, кистообразование, гипертрофию надкостницы, деминерализацию костей черепа. Редкий, но специфичный симптом — образование «бурых» опухолей, чаще формирующихся в различных отделах скелета (бедро, таз, ключицы, ребра, челюсти, череп) [32][109]. Рентгенологическое обследование поясничного и грудного отделов позвоночника в боковой проекции необходимо для исключения или верификации компрессионных переломов тел позвонков [105].

В случае бессимптомного ПГПТ патологические изменения скелета выявляются при снижении МПК по результатам двухэнергетической рентгеновской абсорбциометрии (dual-energy X-ray absorptiometry, DEXA). Всемирная организация здравоохранения (ВОЗ) определяет остеопению как снижение МПК в интервале от 1 до 2,5 стандартных отклонения ниже пикового значения костной массы (Т- критерий в диапазоне от -1,0 до -2,5 SD) и остеопороз как снижение МПК, равное или большее чем 2,5 стандартных отклонения (Т-критерий <-2,5 SD); при исследовании МПК у мужчин моложе 50 лет и женщин до менопаузы используют Z-критерий, значение <-2,0 SD. При ПГПТ, помимо стандартного исследования состояния осевого скелета (поясничного отдела позвоночника и проксимального отдела бедренной кости), необходимо исследовать МПК в периферических костях (дистальная треть предплечья), имеющих преимущественно кортикальное строение и подвергающихся максимальному остеорезорбтивному воздействию ПТГ [48].

УУР С (УДД — 4)

Комментарии: наличие структурных изменений почек является абсолютным показанием к проведению паратиреоидэктомии, таким образом, всем пациентам с ПГПТ рекомендуется выполнение УЗИ и/или КТ почек. КТ считается наиболее информативным методом в диагностике кальцификации почечной паренхимы [110].

Всем пациентам с ПГПТ необходим расчет СКФ. Почечная недостаточность является одним из наиболее тяжелых и малообратимых осложнений и связана с более выраженными клиническими проявлениями, повышением риска смерти, увеличением распространенности артериальной гипертензии [19][61]. Снижение СКФ до ХБП 3-й стадии диагностируется у 17–20% с ПГПТ, при этом часть пациентов могут иметь бессимптомную форму заболевания [64].

Суточная гиперкальциурия характерна в большей мере для ПГПТ с нефролитиазом [111][112]. Радикально выполненная паратиреоидэктомия снижает риск прогрессирования или рецидивирования нефролитиаза, в связи с чем наличие выраженной гиперкальциурии более 10 ммоль/сут (более 400 мг/сут) стало рассматриваться в качестве показания к хирургическому лечению ПГПТ [113].

УУР С (УДД — 4).

## ЛЕЧЕНИЕ ПЕРВИЧНОГО ГИПЕРПАРАТИРЕОЗА

Хирургическое лечение — единственный радикальный и эффективный метод лечения ПГПТ.

Хирургическое лечение ПГПТ рекомендуется:

(УУР B (УДД — 3);

(УУР B (УДД — 3);

(УУР C (УДД — 2);

(УУР А (УДД — 2)

(УУР C (УДД — 4)

(УУР C (УДД — 5)

Комментарии: удаление патологически измененной/ых ОЩЖ является единственным радикальным методом лечения ПГПТ. Динамическое наблюдение и медикаментозная терапия менее экономически выгодны даже в случае бессимптомного ПГПТ [144–146]. Хирургическое лечение показано всем пациентам с классическими проявлениями заболевания [118–120]. Преимущества радикального лечения заключаются в нормализации уровня кальция и устранении ассоциированных с гиперкальциемией симптомов, значимом улучшении состояния костной ткани [120–122] и почек [113][123]. К дополнительным преимуществам можно отнести улучшения со стороны сердечно-сосудистой и нейропсихических систем, заболеваний желудочно-кишечного тракта, однако это требует подтверждения в крупных рандомизированных контролируемых исследованиях [147]. Хирургическое лечение может быть рекомендовано в случае бессимптомного ПГПТ и в отсутствие показаний к паратиреоидэктомии при желании самого пациента, необходима оценка соотношения риска/пользы от операции. Результаты рандомизированных исследований свидетельствуют об улучшении качества жизни пациентов с бессимптомным ПГПТ после хирургического лечения [52][133][148].

Паратиреоидэктомия рекомендуется всем лицам моложе 50 лет, включая детей. В случае отказа от оперативного лечения пациентам моложе 50 лет потребуется более длительный период наблюдения, что ассоциировано с увеличением риска развития специфических осложнений заболевания и финансовых затрат.

Показатели эффективности хирургического лечения достигают 95–98% с частотой послеоперационных осложнений 1–2% при условии выполнения операции опытными хирургами. Показатели смертности при хирургическом лечении ПГПТ низкие. К наиболее серьезным послеоперационным осложнениям относятся парез возвратного гортанного нерва, транзиторная или стойкая гипокальциемия, кровотечение, отсутствие ремиссии заболевания [147]. Отсутствие эффекта после выполнения хирургического вмешательства, как правило, наблюдается в случае синдрома множественных эндокринных неоплазий, атипичного расположения образования ОЩЖ, рака ОЩЖ, отсутствия опыта у хирурга.

УУР C (УДД — 5)

УУР В (УДД — 3)

Комментарии: не так давно наиболее распространенными хирургическими вмешательствами при ПГПТ были «ревизионные операции» — односторонняя и двусторонняя ревизии шеи. Выбор таких объемов хирургического лечения был обусловлен низкой эффективностью топической диагностики и отсутствием методов интраоперационного контроля. Цель таких операций — не только удалить пораженную ОЩЖ, но и предотвратить возможные рецидивы ПГПТ. Однако любые «ревизионные операции» предполагали большую травматичность и риск интраоперационных осложнений [168 –171]. Кроме того, «ревизионные операции» приводили к большей частоте послеоперационного гипопаратиреоза (до 50%). После появления более совершенных методов топической диагностики, позволивших значительно улучшить результаты локализации пораженных ОЩЖ, основной операцией при ПГПТ и солитарном поражении стала селективная паратиреоидэктомия. При этой операции хирург удаляет только пораженную ОЩЖ, опираясь на данные предоперационного обследования [147]. Остальные железы не осматриваются и не травмируются. Селективная паратиреоидэктомия характеризуется высокой эффективностью (95–98%) и низким риском послеоперационных осложнений (1–3%) [149–152].

УУР С (УДД — 4)

Комментарии: при отсутствии четких топических данных о расположении измененной ОЩЖ, наличии множественного поражения ОЩЖ или отсутствии адекватного снижения интраоперационного иПТГ, а также пациентам с литий-индуцированным гиперпаратиреозом рекомендована двусторонняя ревизия шеи с удалением пораженных ОЩЖ [156–158].

УУР А (УДД — 2)

Комментарии: интраоперационное исследование уровня иПТГ до и через 15 минут после удаления образования позволяет оценить радикальность проведенного вмешательства. Снижение уровня иПТГ позволяет хирургу закончить операцию и не проводить ревизию других областей. И наоборот, сохранение исходно высокого уровня иПТГ свидетельствует о сохранении источника(-ов) гиперсекреции ПТГ и требует проведения ревизии с целью их поиска [159–163]. Общая точность метода составляет около 80% [164], как и в случае предоперационной топической диагностики, наилучшие результаты отмечаются при солитарном образовании ОЩЖ (87–99%). При множественном поражении точность интраоперационного определения иПТГ снижается до 58% [164][165].

УУР В (УДД — 3)

Комментарии: после паратиреоидэктомии частота послеоперационной гипокальциемии может варьировать в широком диапазоне, от 5 до 47%. Снижение уровня кальция ниже референсного диапазона может быть обусловлено длительной супрессией нормальных ОЩЖ активной паратиромой, послеоперационным отеком оставшихся ОЩЖ или синдромом «голодных костей». Как правило, снижение уровня кальция ниже референсного диапазона носит транзиторный характер и может корректироваться в амбулаторном порядке [84][154][166–169]. Частота хронического гипопаратиреоза после первичного хирургического лечения крайне низкая, по данным различных исследований, составляет 0–3,6% [154][168][174]. Более низкий уровень кальция крови и более выраженная симптоматика отмечаются у пациентов, перенесших билатеральную ревизию шеи, по сравнению с лицами после минимально инвазивной паратиреоидэктомии [170][171].

Синдром «голодных костей», приводящий к выраженной гипокальциемии, как правило, развивается в раннем послеоперационном периоде у пациентов с тяжелыми костными проявлениями ПГПТ. К основным факторам риска развития синдрома относят пожилой возраст пациентов, рентгенологически верифицированные поражения костной ткани. Другими прогностически неблагоприятными факторами считаются вес и размер аденомы ОЩЖ. Данных об эффективной профилактике данного состояния представлено недостаточно [175].

Лечение препаратами витамина D и кальция рекомендовано пациентам с наличием симптомов гипокальциемии и/или снижением уровня альбумин-скорректированного кальция менее 2,1 ммоль/л (Са++ менее 1,0 ммоль/л) [176–178]. Для устранения гипокальциемии, которая может сохраняться в течение нескольких месяцев после успешной паратиреоидэктомии, необходимы адекватная коррекция дефицита магния и нормализация обмена костной ткани. Предполагается, что предоперационное лечение бисфосфонатами (M05BA) снижает риски послеоперационной гипокальциемии, но на данный момент нет проспективных исследований, посвященных этой проблеме [84][175].

## КОНСЕРВАТИВНОЕ ЛЕЧЕНИЕ

В настоящее время эквивалента хирургическому лечению ПГПТ не существует, поскольку ни один из применяемых препаратов не позволяет достичь равного эффекта по отношению к нормализации лабораторных показателей и улучшению МПК по сравнению с паратиреоидэктомией. Консервативное лечение ПГПТ с использованием медикаментозных препаратов в первую очередь направлено на коррекцию гиперкальциемии и профилактику гиперкальциемических кризов, предупреждение низкотравматичных переломов.

-уровень альбумин-скорректированного кальция крови — 2–4 раза в год;

-уровень креатинина крови с расчетом СКФ (СКD-EPI) — 1 раз в 6 мес;

-исследование уровня иПТГ — 1 раз в 6 мес;

-исследование уровня кальция в моче (суточный анализ) — 1 раз в 6 мес;

-УЗИ почек 1 раз в год (при необходимости КТ забрюшинного пространства);

-рентгеноденситометрия поясничного отдела позвоночника, рентгеноденситометрия проксимального отдела бедренной кости, рентгеноденситометрия лучевой кости; рентгенография грудного и поясничного отдела позвоночника в боковой проекции при подозрении на переломы тел позвонков (снижение в росте, появление болей в спине).

УУР C (УДД — 5).

Комментарии: предложения по динамическому наблюдению пациентов с бессимптомным ПГПТ основываются на результатах исследований, посвященных оценке и сравнению естественного течения заболевания с хирургической тактикой [15][68][118][131][133][136][179][180]. В настоящее время максимальный период наблюдения пациентов с ПГПТ без хирургического лечения составляет 15 лет [180]. Появление одного или более показаний для хирургического лечения за весь период наблюдения зафиксировано у 37% пациентов [180]. Данные по естественной прогрессии нПГПТ остаются ограниченными. В то время как одни исследователи сообщали о высоких показателях прогрессии в виде развития гиперкальциемии, нефролитиаза, гиперкальциурии, переломов и снижения МПК в течение небольшого периода наблюдения (22–41%) [15][68], другими авторами были получены противоположные результаты, свидетельствующие о низком риске ПГПТ-ассоциированных осложнений в данной когорте больных [10][182].

Всем пациентам рекомендуется диета с умеренным потреблением кальция и увеличением потребления жидкости до 1,5–2,0 л в сутки [183]. Резкое ограничение кальция в рационе необходимо в случае выраженной гиперкальциемии более 3 ммоль/л [184][185].

Имеются данные об использовании тиазидов в когорте пациентов с ПГПТ с целью коррекции гиперкальциурии и профилактики нефролитиаза/нефрокальциноза, нарушения почечной функции. Уменьшение суточной экскреции кальция на фоне минимальных и среднетерапевтических доз гидрохлоротиазида** у пациентов с ПГПТ не приводило к повышению показателей кальциемии [186][187]. Однако имеются свои ограничения в данной терапии.

-при отказе пациента от хирургического лечения;

-при наличии противопоказаний к хирургическому лечению (тяжелые сопутствующие заболевания).

УУР C (УДД — 5)

УУР В (УДД — 2)

Комментарии: антирезорбтивная терапия показана пациентам с ПГПТ при снижении МПК в лучевой кости, проксимальном отделе бедра или поясничном отделе позвоночника ≤-2,5 SD по Т-критерию у женщин в постменопаузе и мужчин старше 50 лет по результатам рентгенденситометрии и/или при наличии низкоэнергетических переломов при невозможности выполнения хирургического лечения [193].

Бисфосфонаты (M05BA) снижают активность остеокластов, усиливая их апоптоз, и замедляют резорбцию кости [198]. На основании сходства патогенетических механизмов развития постменопаузального остеопороза и костных нарушений при ПГПТ было проведено несколько сравнительных исследований эффективности и безопасности пероральных бисфосфонатов (M05BA) (алендроновая кислота**) у больных ПГПТ. Данные о долгосрочной эффективности бисфосфонатов (M05BA) в отношении риска переломов ограничены отдельными исследованиями [199–201].

По результатам рандомизированного плацебо-контролируемого двойного слепого исследования 3-й фазы DENOCINA использование деносумаба** в дозе 60 мг 1 раз в 6 мес эффективно в отношении улучшения МПК и снижения скорости метаболизма костной ткани у пациентов с ПГПТ независимо от сочетанной терапии цинакальцетом** [196]. Деносумаб** имеет преимущество по сравнению с бисфосфонатами (M05BA) в отношении прироста МПК в кортикальной кости [202].

Цинакальцет** снижает уровень сывороточного кальция и иПТГ за счет повышения чувствительности CaSR к концентрации внеклеточного кальция. Использование цинакальцета** приводит к стойкой нормализации показателей кальциемии у 70–80% пациентов с ПГПТ [203]. Эффект может сохраняться в течение 5 лет, однако уровень сывороточного кальция возвращается к исходным значениям сразу после прекращения терапии. Данный препарат не влияет напрямую на показатели МПК и маркеры костной резорбции [203]. Убедительных данных о воздействии препарата на симптомы гиперкальциемии, нефролитиаз или качество жизни в литературе не представлено.

УУР A (УДД — 2)

УУР A (УДД — 2)

Комментарии: для устранения вторичного повышения иПТГ на фоне дефицита витамина D рекомендуется поддерживать показатели 25(ОН)D на уровне не менее 20 нг/мл (50 нмоль/л), оптимальным считается уровень >30 нг/мл (75 нмоль/л) [17][206][207]. После успешной паратиреоидэктомии у ряда пациентов сохраняется повышенный уровень иПТГ, что может быть обусловлено имеющимся дефицитом/недостаточностью витамина D [208–211]. Достижение оптимального уровня витамина D после радикальной операции способствует нормализации уровня иПТГ, адекватной реабсорбции кальция и улучшению показателей МПК [126][205].

УУР В (УДД — 3)

## ГИПЕРКАЛЬЦИЕМИЧЕСКИЙ КРИЗ

УУР С (УДД — 4)

Комментарии: резкое повышение уровня кальция в сыворотке крови может приводить к развитию гиперкальциемического криза и провоцируется тяжелыми интеркуррентными состояниями: развитием инфекционных заболеваний, переломов, длительной иммобилизацией, приемом антацидных средств [213][214]. Гиперкальциемический криз, как правило, отмечается при повышении уровня альбумин-скорректированного кальция более 3,5 ммоль/л (Са++ более 1,8 ммоль/л)* и характеризуется симптомами полиорганной дисфункции, включая поражение ЖКТ (анорексия, тошнота, неукротимая рвота с развитием дегидратации, острая боль в животе, острый панкреатит), почек (олигурия, острая почечная недостаточность, почечная колика), сердечно-сосудистой системы (нарушение проводимости и сердечного ритма, укорочение интервала Q–T), нервной системы (миалгии, мышечная слабость, спутанность сознания, ступор, кома до 40% случаев) [213–219].

Своевременное лечение пациентов с гиперкальциемическим кризом имеет решающее значение, так как это состояние ассоциировано с высоким уровнем летального исхода [213][215]. Гиперкальциемический криз при ПГПТ является показанием для проведения операции в срочном порядке. Консервативная тактика в отношении пациентов с тяжелой гиперкальциемией должна рассматриваться в качестве «подготовки» к оперативному лечению и, по возможности, проводиться в условиях отделения реанимации и интенсивной терапии [213–219]. Для уменьшения риска общей анестезии используются консервативные методы, направленные на коррекцию гиповолемии, стимулирование почечной экскреции кальция и торможение ускоренной резорбции костной ткани (табл. 3). Оптимальные сроки для паратиреоидэктомии у пациентов с гиперкальциемическим кризом остаются дискутабельными. Основываясь на результатах топических методов диагностики, селективная паратиреоидэктомия может рассматриваться в качестве оптимальной тактики для большинства пациентов. Тем не менее тяжелая гиперкальциемия/гиперкальциемический криз в 4,5–12% случаев могут быть обусловлены наличием карциномы ОЩЖ.

**Table:** Таблица 3. Консервативное лечение тяжелой гиперкальциемии

Препарат	Функция	Предостережения
Отмена кальций-повышающих лекарственных средств
Натрия хлорид** 300–500 мл/ч (2–6 л/сут, ежедневно 1–3 дня) до полного возмещения дефицита жидкости и восстановления диуреза	Увеличивает фильтрацию и выделение кальция.Понижает кальциемию на 0,25–0,75 ммоль/л	Избегать гипергидратации у пациентов с сердечно-сосудистой патологией
#фуросемид** 20–40 мг внутривенно по необходимости (диурез не менее 200–300 мл/ч) [221]	Целесообразно назначение у пациентов с риском кардиогенного отека легких. В этом случае, при достижении эуволемии, активная регидратация (например, 3 л 0,9% физиологического раствора в течение 24 ч) должна быть сбалансирована внутривенным введением фуросемида для поддержания водного баланса.Также ингибирует реабсорбцию кальция в дистальных канальцах	Гипокалиемия, обезвоживание,если внутрисосудистый объем жидкости не восстановлен
#деносумаб** 60 мг п/к [222]	Моноклональное человеческое антитело к лиганду рецептора активатора ядерного фактора каппа В (RANKL), антирезорбтивный эффект,возможность применения при ХБП	Гипокальциемия, воспаление подкожножировой клетчатки в месте введения (редко)
Бисфосфонаты (M05BA)	Подавляют функцию и активность остеокластов и резорбцию кости	Нефротоксичны, противопоказаны при СКФ менее 35 мл/мин/1,73 м2, снижают уровень кальция, уровень фосфора. Максимальные эффекты — в течение 72 ч
Ибандроновая кислота 3 мг в/в болюсно в течение 15–30 с [223]
Цинакальцет** 30–360 мг/сутвнутрь ежедневно. Начальная доза 30 мг/сут с последующим титрованием каждые 2–4 нед до достижения оптимального уровня кальция (нормокальциемия или снижение альбумин-скорректированного кальция до уровня, не превышающего 0,25 ммоль/л (1 мг/дл) относительно верхней границы референсного диапазона)	Агонист кальций-чувствительного рецептора,возможность применения при ХБП	Гипокальциемия, тошнота, рвота

## МЕДИЦИНСКАЯ РЕАБИЛИТАЦИЯ

Специфических реабилитационных мероприятий в отношении пациентов с ПГПТ не разработано.

## ПРОФИЛАКТИКА

УУР С (УДД — 4)

Комментарии: как правило, активное восстановление костного метаболизма, существенный прирост МПК (в среднем 5–10% за год) в зависимости от выраженности костных потерь и возраста пациента происходит в течение первых 2 лет после успешно проведенной паратиреоидэктомии. В этот период не требуется дополнительное лечение, за исключением поддержания адекватного уровня 25(ОН) витамина D, назначения альфакальцидола**/кальцитриола** и препаратов кальция [118][129][133][135][180][224][225].

УУР С (УДД — 5)

## НАСЛЕДСТВЕННЫЕ ФОРМЫ ПЕРВИЧНОГО ГИПЕРПАРАТИРЕОЗА

До 10% случаев ПГПТ имеют наследственную природу (табл. 4). Наследственные формы ПГПТ характеризуются манифестацией в молодом возрасте и отсутствием гендерных отличий [5]. Молекулярно-генетическая диагностика позволяет своевременно поставить диагноз и начать скрининг компонентов синдрома и их лечение, что особенно важно для бессимптомных заболеваний.

**Table:** Таблица 4. Наследственные синдромы, ассоциированные с развитием первичного гиперпаратиреоза

	МЭН-1	МЭН-2А1	МЭН-4	HPT-JT	FIHP2
OMIM#	131100	171400	610755	145001	145000
Тип наследования	АД	АД	АД	АД	АД
Локус на хромосоме	11q13	10q11.2	12p13.1	1q31.2	2p130.3-14
Мутация в гене	MEN1	RET	CDKN1B	HRPT2/CDC73	MEN1, CDC73/HRPT2, CaSR (?)
Кодируемый белок	Менин	RET	р27(Kip1)	Парафибромин	–
Другие проявления	Опухоли аденогипофиза и эндокринной части поджелудочной железы, другие нейроэндокринные и неэндокринные опухоли	Медуллярный рак щитовидной железы и феохро-моцитома	Опухоли аденогипофиза, нейроэндокринные опухоли ЖКТ и легких, образования поджелудочной железы и надпочечников и др.	Оссифицирующие фибромы нижней челюсти, опухоли почек и матки	–

-манифестация ПГПТ у пациентов моложе 40 лет;

-множественное поражение ОЩЖ в любом возрасте;

-персистенция/рецидив ПГПТ;

-наличие 2-х и более МЭН-ассоциированных образований (опухолей ОЩЖ, нейроэндокринных опухолей (НЭО) поджелудочной железы (ПЖ), аденомы гипофиза);

-родственники первой линии родства носителя мутации в гене MEN1 даже в случае отсутствия симптомов;

-гастринома или НЭО поджелудочной железы в любом возрасте при наличии 2 и более МЭН-ассоциированных опухолей, не относящихся к классической триаде МЭН-1.

УУР C (УДД — 5)

Комментарии: МЭН-1 синдром — заболевание с аутосомно-доминантным типом наследования, обусловленное герминальными мутациями в гене MEN1. MEN1 является геном-супрессором опухолевого роста и кодирует белок менин, регулирующий различные функции клеточного и геномного гомеостаза. Корреляций между генотипом и фенотипом заболевания не выявлено [5]. Распространенность синдрома составляет 2–3 случая на 100 000 человек. Синдром МЭН-1 характеризуется сочетанным развитием опухолей ОЩЖ (в 95% случаев), аденогипофиза (15–55%) и островкового аппарата поджелудочной железы (30–80%), представляющими собой «классическую триаду» заболевания. Реже диагностируются опухоли надпочечников, НЭО тимуса, легких и желудочно-кишечного тракта, ангиофибромы, коллагеномы, липомы, лейомиомы, менингиомы и другие [228–234]. Большинство новообразований в рамках синдрома являются доброкачественными, однако сохраняется высокий риск злокачественной прогрессии этих опухолей.

ПГПТ, как правило, является первым проявлением синдрома (до 75%) [228][235][236], при этом распространенность МЭН-1 среди пациентов ПГПТ составляет 1–18% [235]. Дебют ПГПТ при МЭН-1 обычно приходится на период между 20 и 25 годами [231][235][237], при этом пенетрантность заболевания при МЭН-1 с возрастом достигает 90–100% [238]. Поражение ОЩЖ чаще бывает множественным и как правило, не является одномоментным (множественные гиперплазии или аденомы). В 20% случаев возникает поражение дополнительных и эктопированных ОЩЖ с возможной локализацией в тимусе, щитовидной железе, переднем средостении и, иногда, в перикарде [235][239][240]. Клиническая картина представлена как бессимптомной (асимптомная гиперкальциемия), так и симптомной формой ПГПТ. Для ПГПТ в рамках МЭН-1 синдрома характерна большая частота рецидивов по сравнению со спорадическими формами [228][235][241].

Результаты пилотных исследований российской популяции свидетельствуют о смещении возраста манифестации ПГПТ при МЭН-1 на 3-ю декаду жизни, в связи с чем рекомендуемый возрастной порог для проведения генетического исследования составляет до 40 лет [240][242].

УУР А (УДД — 2)

Комментарии: в связи с полигландулярным поражением ОЩЖ при МЭН-1 синдроме основным методом лечения ПГПТ остается двусторонняя ревизия шеи с субтотальным (≥3,5 желез) или тотальным удалением ОЩЖ с аутотрансплантацией участка наименее измененной в плечелучевую мышцу, характеризующейся наименьшим риском рецидива заболевания [243–247]. Частота развития хронического гипопаратиреоза, в том числе тяжелого течения, значимо выше в группе тотальной резекции четырех ОЩЖ, чем в группе субтотальной паратиреоидэктомии [243–245].

УУР С (УДД — 4)

Комментарии: необходимые исследования и кратность их проведения представлены в таблице 5.

 

**Table:** Таблица 5. Биохимический и рентгенологический скрининг у пациентов с синдромом множественных эндокринных неоплазий 1 типа

Проявление МЭН-1	Ежегодное лабораторное обследование	Методы топической диагностики
Образования ОЩЖ	Альбумин-скорректированный кальций/ Са++, иПТГ	При лабораторном подтверждении впервые выявленного ПГПТ — УЗИ и ОФЭКТ/КТ ОЩЖ.При необходимости КТ с контрастом
Гастринома	Нет, пока отсутствуют топические данные или клинические симптомы заболевания (гастрин, экспресс PH-метрия)	Нет, при клинических симптомах заболевания:МСКТ/МРТ;для подтверждения диагноза и локализации — соматостатин-рецепторная сцинтиграфия;ПЭТ-КТ с туморотропным радиофармацевтическим диагностическим средством (68Ga-DOTA-TATE/NOC/TOC и др.)
Инсулинома	Нет, пока отсутствуют топические данные или клинические симптомы заболевания (инсулин, проинсулин, гликемия натощак, 3-дневная проба с голоданием)	Нет, при клинических симптомах заболевания:МСКТ/МРТ; для подтверждения диагноза и локализации;соматостатин-рецепторная сцинтиграфия;ПЭТ-КТ с туморотропным радиофармацевтическим диагностическим средством (68Ga-DOTA-TATE/NOC/TOC и др.)
Другие НЭО ПЖ	Нет, пока отсутствуют топические данные или клинические симптомы заболевания (хромогранин А, ПП, глюкагон, ВИП)	МРТ/КТ брюшной полости 1 раз в 2 года (ежегодно).При необходимости — эндоУЗИ, соматостатин-рецепторная сцинтиграфия; ПЭТ-КТ с туморотропным радиофармацевтическим диагностическим средством (68Ga-DOTA-TATE/NOC/TOC и др.)
Аденомы гипофиза	Пролактин, ИФР-1	МРТ гипофиза 1 раз в 3 года
Карциноид легкого и тимуса	Нет, как правило, гормонально-неактивны, но со злокачественным потенциалом	КТ грудной полости 1 раз в 2-3 года.При необходимости — ПЭТ КТ с 18F-ФДГ; cоматостатин-рецепторная сцинтиграфия;ПЭТ-КТ с туморотропным радиофармацевтическим диагностическим средством (68Ga-DOTA-TATE/NOC/TOC и др.)
Образования надпочечников	Нет, при впервые выявленной инциденталоме надпочечника или появлении клинических признаков гормональной активности (активность ренина плазмы, альдостерон, подавляющий тест с 1 мг дексаметазона или кортизол в вечерней слюне или кортизол в суточной моче, метанефрины в суточной моче)	МРТ/КТ забрюшинного пространства 1 раз в 2 года (ежегодно).При необходимости сцинтиграфия с 123I-MIBG;ПЭТ-КТ с туморотропным радиофармацевтическим диагностическим средством (18F-DOPA и др.);соматостатин-рецепторная сцинтиграфия

 

-наличие медуллярного рака щитовидной железы в анамнезе или на момент диагностики ПГПТ;

-наличие феохромоцитомы в анамнезе или на момент диагностики ПГПТ;

-данные о наличии медуллярного рака щитовидной железы и/или феохромоцитомы и/или синдрома МЭН-2А у родственников первой линии родства.

УУР C (УДД — 4)

Комментарии: синдром МЭН-2А проявляется развитием медуллярного рака щитовидной железы (90–100%), феохромоцитомы (50%), ПГПТ (20–30%) и обусловлен герминальными мутациями в прото-онкогене RET с усилением его функции [250–252]. Мутация гена RET в эмбриональных клетках приводит к экспрессии патологически измененного сверхактивного RET-протеина в нейроэндокринных тканях, что приводит к неконтролируемой клеточной пролиферации. В случае МЭН-2А только в 5% ПГПТ предшествует развитию других компонентов, в подавляющем большинстве заболевание выявляется во время операции по поводу медуллярного рака щитовидной железы, или спустя годы после нее. ПГПТ характеризуется более легким течением, чем при синдроме МЭН-1, и в 42–84% случаев протекает бессимптомно [239][253][254]. Средний возраст на момент диагностики составляет 35–39 лет [250]. Чаще, чем при МЭН 1 типа (27–48%) поражается только одна ОЩЖ, и только в 8% случаев выявляются парные аденомы или гиперплазии [255][256].

УУР С (УДД — 4)

Комментарии: единого мнения по поводу объема хирургического лечения ПГПТ в рамках МЭН-2А в настоящее время не существует. Хирургические варианты лечения ПГПТ включают: резекцию визуально измененных ОЩЖ; субтотальную паратиреоидэктомию; тотальную паратиреоидэктомию с гетеротопической аутотрансплантацией [147][257][258]. Cубтотальная и тотальная паратиреоидэктомия с гетеротопической аутотрансплантацией оправдана в случае множественного поражения ОЩЖ [254]. Актуальную проблему представляет высокий риск тяжелого хронического гипопаратиреоза, обусловленного, прежде всего, проведением расширенной тиреоидэктомии по поводу медуллярного рака щитовидной железы. У пациентов с МЭН-2А, перенесших профилактическую тиреоидэктомию, уровни кальция сыворотке крови должны обязательно определяться на этапе дооперационной диагностики. Кроме того, перед проведением операций как по поводу медуллярного рака щитовидной железы, так и ПГПТ, необходимо исключение диагноза феохромоцитомы [252].

-наличие родственника первой линии родства с синдромом HPT-JT;

-карцинома ОЩЖ;

-наличие оссифицирующих фибром нижней/верхней челюсти;

-наличие поликистоза почек, опухолей почек, опухолей матки.

УУР С (УДД — 4)

Комментарии: cиндром HPT-JT — редкое аутосомно-доминантное заболевание, характеризуется развитием ПГПТ, оссифицирующих фибром нижней и/или верхней челюсти в 30–40%, опухолями матки (лейомиомы, гиперплазия эндометрия, аденосаркомы, аденофибромы, множественные аденоматозные полипы) у 57,3% больных женщин, реже поражением почек (гамартомы, поликистоз почек, опухоли Вильмса, аденокарциномы) в 13,3% случаев. Причина — мутация гена CDC73 (HRPT2), кодирующего парафибромин [259–263].

ПГПТ — основное проявление HPT-JT, выявляется приблизительно в 95% случаев [259][262]. Частота ПГПТ увеличивается с возрастом, хотя заболевание манифестирует в раннем молодом возрасте [264]. ПГПТ в рамках HPT-JT обычно обусловлен единичной доброкачественной аденомой ОЩЖ, кистозной или с атипичными гистологическими характеристиками. В отличие от других наследственных вариантов ПГПТ, распространенность карцином ОЩЖ в рамках HPT-JT выше и достигает 10–21,6% [259]. Мультигландулярное поражение диагностируется редко при первичной операции (20% случаев), вторая аденома ОЩЖ может возникнуть метахронно спустя годы или десятилетия после возникновения первичной опухоли (23,9% случаев). Заболевание может протекать в бессимптомной форме, карциномы ОЩЖ часто протекают с гиперкальциемическими кризами [259].

Оптимальный хирургический подход при ПГПТ в рамках HPT-JT пока не разработан. В последнее время чаще предлагается селективная ПТЭ при поражении одной ОЩЖ и отсутствии подозрения на злокачественность. В случае подозрения на рак ОЩЖ (большие образования ОЩЖ с инфильтративным ростом, крайне высокие показатели кальция и иПТГ) предпочтительно выполнение резекции единым блоком с удалением опухоли ОЩЖ, ипсилатеральной половины щитовидной железы, окружающей клетчатки, а также любой спаянной с опухолью ткани для предотвращения повреждения опухоли и диссеминации [259][260].

Другие наследственные синдромы, в рамках которых может возникать ПГПТ, встречаются крайне редко, что не позволяет сформировать клинические рекомендации по их диагностике и лечению.

При наличии клинических признаков синдрома МЭН-1 и в отсутствие мутации в гене MEN1 можно заподозрить синдром множественных эндокринных неоплазий 4 типа (МЭН-4) — «МЭН-1-подобное» состояние, фенокопию МЭН-1. Обусловлен мутацией в гене CDKN1B. Наиболее частые компоненты синдрома — ПГПТ и аденомы гипофиза. Другие проявления: НЭО бронхов и желудка, гастринома, папиллярный рак щитовидной железы, объемные образования поджелудочной железы и надпочечников. Лечение — удаление единичной аденомы ОЩЖ либо субтотальная паратиреоидэктомия [265].

При наличии в семье нескольких членов с ПГПТ и отсутствии других эндокринных и неэндокринных опухолей можно заподозрить семейный изолированный гиперпаратиреоз FIHP. Это редкое аутосомно-доминантное заболевание, характеризующееся одиночным или множественными поражениями ОЩЖ в отсутствие специфических проявлений других синдромов (МЭН-1, HPT-JT, FHH). Оптимальный хирургический подход при ПГПТ в рамках FIHP не разработан. При поражении одной ОЩЖ может проводиться селективная паратиреоидэктомия, а при множественном поражении рекомендуется субтотальная резекция. При наличии мутаций MEN1 и CDC73 — лечение как при МЭН-1 и HPT-JT [7].

## РАК ОКОЛОЩИТОВИДНЫХ ЖЕЛЕЗ

Рак (карцинома) ОЩЖ — редкая патология, характеризующаяся тяжелым течением и высокой смертностью вследствие выраженной гиперкальциемии. Часто диагноз карциномы ОЩЖ удается установить спустя годы от начала заболевания при развитии рецидива. В структуре ПГПТ рак ОЩЖ занимает менее 1% [266].

-сочетание повышения альбумин-скорректированного кальция более 3 ммоль/л и одного из продольных размеров образования ОЩЖ более 3 см [267];

-повышение уровня Са++ крови более 1,6–1,7 ммоль/л [268–270];

-симптомы «масс-эффекта» при отсутствии других объемных образований и операций в области шеи в анамнезе [270–273].

УУР С (УДД — 4)

Комментарии: основные клинические, лабораторно-инструментальные характеристики рака ОЩЖ резюмированы в табл. 6. Предоперационная топическая диагностика при злокачественных новообразованиях ОЩЖ соответствует общим принципам топической диагностики при ПГПТ.

**Table:** Таблица 6. Сравнительная таблица клинических и биохимических проявлений злокачественного и доброкачественного новообразований околощитовидных желез [267][272][274][275]

Признаки	Аденома ОЩЖ	Карцинома ОЩЖ
Соотношение ж/м	4/1	1/1
Уровень общего кальция крови, ммоль/л	2,7–2,9	>3
Са++, ммоль/л	<1,6	>1,7
Уровень иПТГ, пг/мл	<2 (N)*	>3–10 (N)
Манифестация заболевания	50–60 лет	40–50 лет
Пальпируемое образование	<2%	30–76%
Фиброзно-кистозный остеит	5%	40–75%
Нефролитиаз	10–15%	40%
Сочетание костной и почечной патологии	редко	40–50%
Асимптомное течение	60–80%	5%
Объем образования, см	<3	>3
УЗИ признаки	ИзоэхогенностьРовный контурОднородная структура	Гипоэхогенность Неровный контурНеоднородная структура

УУР С (УДД — 4)

Комментарии: методом выбора лечения рака ОЩЖ остается хирургическое удаление опухоли «единым блоком» со смежными тканями. Основная цель — избежать разрыва капсулы карциномы во время операции, что является неблагоприятным прогностическим фактором [267][276][277]. Для рака ОЩЖ характерна плотная консистенция опухоли, спаянность с окружающими тканями, а также наличие измененных лимфатических узлов [267][276][277]. При ретроспективной постановке диагноза и в случае достижения ремиссии заболевания после МИП вопрос о повторной операции должен решаться в индивидуальном порядке.

УУР С (УДД — 5)

УУР С (УДД — 4)

УУР С (УДД — 5)

Комментарии: для карциномы ОЩЖ характерны медленный рост опухоли и поздние метастазы в легкие, шейные лимфоузлы, печень и кости. Реже встречаются метастазы в плевру, поджелудочную железу и перикард [107]. Отсутствие ремиссии, так же, как и рецидив ПГПТ, устанавливается при сочетании гиперкальциемии и повышенного уровня иПТГ. Повышение иПТГ при нормокальциемии или гипокальциемии следует дифференцировать с вторичным гиперпаратиреозом. В случае доказанного метастаза ОЩЖ рекомендуется его хирургическое удаление, когда это возможно. Резекция отдаленных метастазов увеличивает выживаемость пациентов в результате достижения нормокальциемии (основная причина смерти пациентов с раком ОЩЖ — гиперкальциемия) [123]. При отсутствии ремиссии или рецидиве ПГПТ после хирургического лечения у пациента с раком ОЩЖ с целью поиска метастатических очагов рекомендовано применение стандартных методов инструментальной диагностики. Поиск вторичных очагов (метастазов), наиболее часто локализующихся в костях, осложнен наличием фиброзно-кистозного остеита, который нередко является причиной ложноположительного заключения о метастатическом поражении костной ткани [281–283].

УУР С (УДД — 5)

УУР С (УДД — 4)

УУР С (УДД — 3)

Комментарии: тяжелая гиперкальциемия, часто сопровождающая рак ОЩЖ, является жизнеугрожающим состоянием, требующим консервативной коррекции в случае отсутствия возможности хирургического удаления гормон-продуцирующих очагов. В этих условиях применяются общие принципы коррекции гиперкальциемии. В ряде случаев пациентов с диссеминированными формами рака ОЩЖ описан положительный опыт применения ингибиторов протеинкиназы [294][295]. Возможность назначения подобного лечения должна обсуждаться и решаться в учреждениях федерального уровня по результатам заключения консилиума.

## ПЕРВИЧНЫЙ ГИПЕРПАРАТИРЕОЗ И БЕРЕМЕННОСТЬ

Выявляемость ПГПТ среди женщин детородного возраста составляет около 8 на 100 000 чел./год, среди беременных — 0,15 до 1,4%. В 67% случаев ПГПТ во время беременности приводит к осложнениям со стороны матери и в 80% к развитию патологии у плода. Летальность плода или новорожденного может достигать 20–30% [296].

УУР С (УДД — 4)

Комментарии: диагноз ПГПТ должен быть заподозрен при выявлении повышения альбумин-скорректированного или Са++, при гипофосфатемии в сочетании с увеличением уровня иПТГ [297][298]. Обследование на предмет гиперпаратиреоза должно проводиться в случае любых метаболических нарушений костной системы, при нетравматических переломах, при рецидивирующей мочекаменной болезни, при стойком панкреатите и рецидивирующих язвах желудка или двенадцатиперстной кишки, при инсипидарном синдроме [297].

УУР С (УДД — 4)

Комментарии: основным методом топической диагностики ПГПТ при беременности является ультразвуковое исследование (УЗИ) шеи. КТ, сцинтиграфия ОЩЖ, МРТ обычно не используются из-за потенциального риска для плода [301][302].

УУР С (УДД — 4)

Комментарии: хирургическое лечение оптимально проводить во II триместре беременности, когда завершен органогенез и риск преждевременных родов по сравнению с III триместром значимо ниже [301][303–305]. Однако отдельные авторы указывают на возможность хирургического удаления образования ОЩЖ независимо от гестационного периода при наличии абсолютных показаний. В случае, если операция может быть отсрочена, хирургическое удаление образования ОЩЖ должно быть выполнено как можно раньше после родоразрешения с целью предупреждения гиперкальциемического криза. В ряде случаев требуется одновременное выполнение кесарева сечения и паратиреоидэктомии [308].

УУР С (УДД — 5)

Комментарии: в случае бессимптомной формы ПГПТ с умеренным повышением уровня кальция допускается консервативное ведение пациенток с соблюдением достаточного питьевого режима и под регулярным контролем показателей фосфорно-кальциевого обмена. При недостаточности витамина D рекомендуется назначение колекальциферола**, так как это препятствует дальнейшему вторичному повышению уровня иПТГ, рекомендуется использование небольших доз (500–1000 МЕ/сут) и частый контроль показателей фосфорно-кальциевого обмена [298][301].

УУР С (УДД — 5)

## ДИФФЕРЕНЦИАЛЬНАЯ ДИАГНОСТИКА ГИПЕРКАЛЬЦИЕМИИ

Целью дифференциальной диагностики ПГПТ с другими состояниями, сопровождаемыми повышением уровня кальция, является определение причины гиперкальциемии, что позволило бы назначить соответствующее лечение, направленное на ликвидацию первичного заболевания. Наиболее частой причиной развития гиперкальциемии является ПГПТ, обуславливающий более 80% случаев повышения уровня кальция крови. Среди госпитализированных пациентов в числе причин гиперкальциемии на первое место выходят злокачественные новообразования легких и почек, гемобластозы (миеломная болезнь, лимфомы, лимфогранулематоз, лейкозы) и составляют 50–60%. Реже повышение уровня кальция отмечается при раке толстого кишечника и предстательной железы. Описаны эктопические новообразования, продуцирующие ПТГ вне ткани ОЩЖ, чаще при раке молочной железы. Саркоидоз ассоциируется с гиперкальциемией в 20%, а с гиперкальциурией — примерно в 40% случаев. Гиперкальциемия может также развиваться вследствие химиотерапии по поводу онкологических заболеваний (20–30%), приводящей к повышению костной резорбции (выраженность эффекта дозозависима).

Полная версия клинических рекомендаций размещена на сайте в рубрикаторе Минздрава РФ https://cr.minzdrav.gov.ru/recomend/88_4.

## ЗАКЛЮЧЕНИЕ

ПГПТ — распространенное эндокринное нарушение, требующее расширенного диагностического поиска и длительного наблюдения пациентов. Активный скрининг кальция направлен на раннее выявление гиперкальциемии как первого диагностического маркера заболевания. Создание единых клинических рекомендаций, основанных на принципах доказательной медицины, — социально значимая инициатива для предотвращения инвалидизации, повышения качества оказания медицинской помощи и жизни пациентов.

## ПРИЛОЖЕНИЕ.МЕТОДОЛОГИЯ РАЗРАБОТКИ КЛИНИЧЕСКИХ РЕКОМЕНДАЦИЙ

**Table:** Таблица 1. Шкала оценки уровней достоверности доказательств (УДД) для методов диагностики (диагностических вмешательств)

УДД	Расшифровка
1	Систематические обзоры исследований с контролем референсным методом или систематический обзор рандомизированных клинических исследований с применением метаанализа
2	Отдельные исследования с контролем референсным методом или отдельные рандомизированные клинические исследования и систематические обзоры исследований любого дизайна, за исключением рандомизированных клинических исследований, с применением метаанализа
3	Исследования без последовательного контроля референсным методом или исследования с референсным методом, не являющимся независимым от исследуемого метода или нерандомизированные сравнительные исследования, в том числе когортные исследования
4	Несравнительные исследования, описание клинического случая
5	Имеется лишь обоснование механизма действия или мнение экспертов

**Table:** Таблица 2. Шкала оценки уровней достоверности доказательств (УДД) для методов профилактики, лечения и реабилитации (профилактических, лечебных, реабилитационных вмешательств)

УДД	Расшифровка
1	Систематический обзор РКИ с применением метаанализа
2	Отдельные РКИ и систематические обзоры исследований любого дизайна, за исключением РКИ, с применением мета-анализа
3	Нерандомизированные сравнительные исследования, в т.ч. когортные исследования
4	Несравнительные исследования, описание клинического случая или серии случаев, исследования «случай-контроль»
5	Имеется лишь обоснование механизма действия вмешательства (доклинические исследования) или мнение экспертов

**Table:** Таблица 3. Шкала оценки уровней убедительности рекомендаций (УУР) для методов профилактики, диагностики, лечения и реабилитации (профилактических, диагностических, лечебных, реабилитационных вмешательств)

УУР	Расшифровка
A	Сильная рекомендация (все рассматриваемые критерии эффективности (исходы) являются важными, все исследования имеют высокое или удовлетворительное методологическое качество, их выводы по интересующим исходам являются согласованными)
B	Условная рекомендация (не все рассматриваемые критерии эффективности (исходы) являются важными, не все исследования имеют высокое или удовлетворительное методологическое качество и/или их выводы по интересующим исходам не являются согласованными)
C	Слабая рекомендация (отсутствие доказательств надлежащего качества (все рассматриваемые критерии эффективности (исходы) являются неважными, все исследования имеют низкое методологическое качество и их выводы по интересующим исходам не являются согласованными)

## ПРИЛОЖЕНИЕ Б.АЛГОРИТМ ДЕЙСТВИЙ ВРАЧА

**Figure:**